# The Applications of Gold Nanoparticle-Initialed Chemiluminescence in Biomedical Detection

**DOI:** 10.1186/s11671-016-1686-0

**Published:** 2016-10-18

**Authors:** Zezhong Liu, Furong Zhao, Shandian Gao, Junjun Shao, Huiyun Chang

**Affiliations:** Stake Key Laboratory of Veterinary Etiological Biology, National Foot-and-Mouth Diseases Reference Laboratory, Lanzhou Veterinary Research Institute, Chinese Academy of Agricultural Sciences, Lanzhou, 730046 China

**Keywords:** Chemiluminescence, Gold nanoparticles, Detection, Biomedicine

## Abstract

Chemiluminescence technique as a novel detection method has gained much attention in recent years owning to the merits of high sensitivity, wider linear ranges, and low background signal. Similarly, nanotechnology especially for gold nanoparticles has emerged as detection tools due to their unique physical and chemical properties. Recently, it has become increasingly popular to couple gold nanoparticles with chemiluminescence technique in biological agents’ detection. In this review, we describe the superiority of both chemiluminescence and gold nanoparticles and conclude the different applications of gold nanoparticle-initialed chemiluminescence in biomedical detection.

## Review

### Introduction

Detection of biological agents plays an indispensable role in biomedicine [1]. In clinical diagnosis, developing highly sensitive and cost-effective detection methods is in high demand for the reason that some clinical samples have very low concentration that usually cannot be detected. Since the advent of radioimmunoassay (RIA) [2], the trace materials could be detected. From then, detection technologies have gained much attention and a few techniques were developed, including fluoroimmunoassay (FIA) and enzyme immunoassay (EIA). The FIA and EIA were regularly used but sometimes cannot meet the clinical demand due to their low sensitivities. The RIA is characterized by great sensitivity but has some drawbacks, including health hazard, short half-life, and environment contamination [3].

In the late 1970s, chemiluminescence immunoassay (CLIA) was first introduced [4]. The mechanism of chemiluminescence (CL) is that some molecules can get the energy from the chemical reaction and be excited to the electronically excited state; the energy is disposed of in the form of light along with the molecules return to ground state [5]. It does not need photoluminescence and the energy comes from chemical interaction, which leads to low background signal [6, 7]. In addition, it also has some merits including ultrasensitivity, high signal-to-noise ratio, low cost, and wide linear dynamic ranges [8, 9]. All of those advantages make CL to be a powerful detection method.

Nanobiotechnology is the product of biology and nanotechnology, which develops rapidly and shows a significant future promise [10]. Almost all the areas of biomedicine such as bioimaging [11, 12], drug delivery [13, 14], oncotherapy [15, 16], and clinical diagnosis [17] have the shadow of nanomaterials. Among the nanomaterials including magnetic nanoparticles, quantum dots, graphene oxide, carbon nanotubes, and gold nanoparticles (AuNPs), the AuNP is the most widely used.

**Fig. 1:**
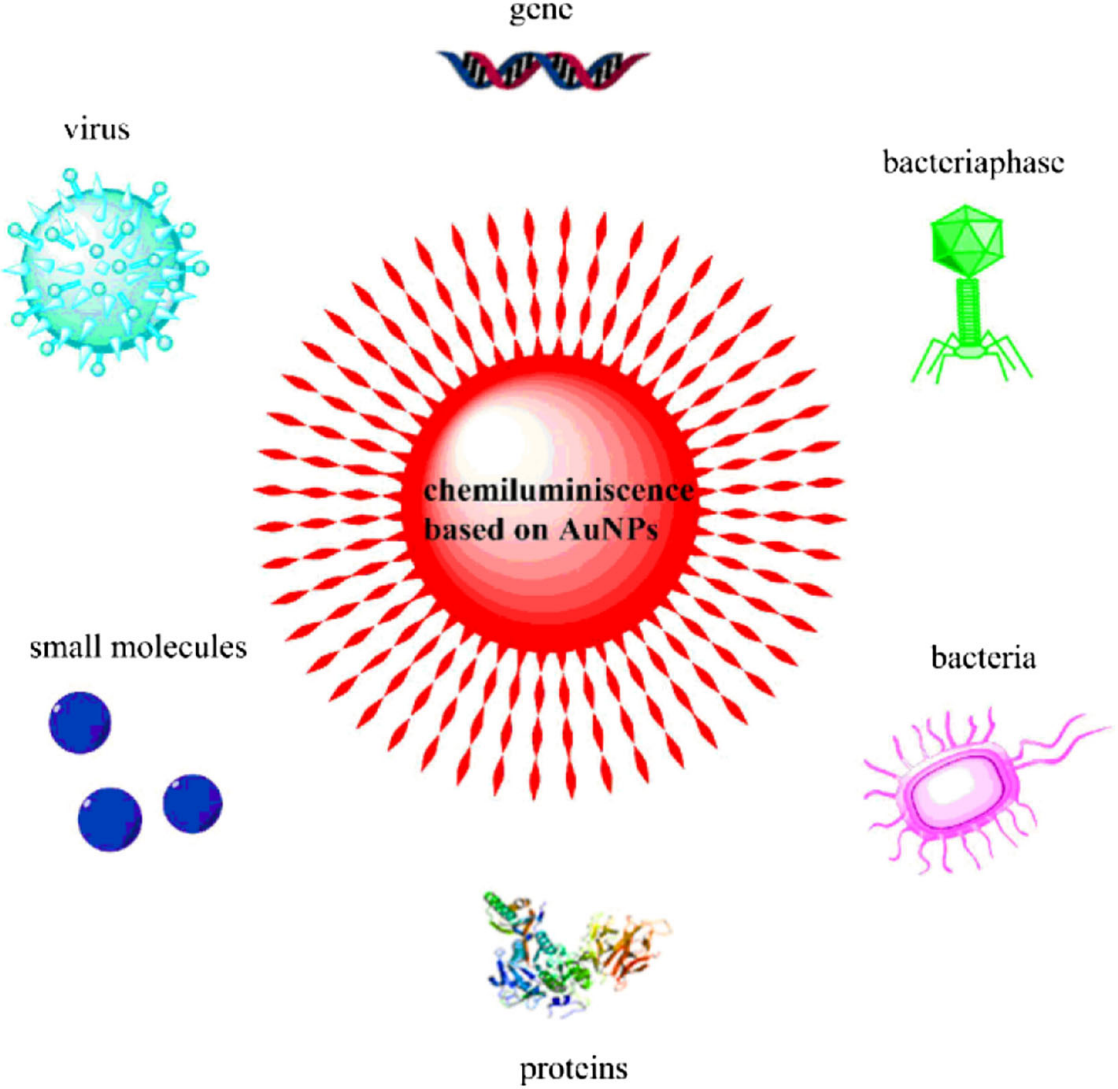
Examples of CL based on AuNPs for detection of biomolecules

Nanotechnology, especially for AuNPs, which offers a wealth of particular characteristics suited for diagnosis, has becoming a promising strategy to enhance the CL sensitivity. In recent years, the CL detection method based on AuNPs has continuously emerged. The detection limit and detection time has been improved extremely. This AuNP-initialed CL detection method has been applied in different fields such as for detection and diagnosis of microorganism, protein, nucleic acids, pharmaceutical molecules, and so many small molecules (Fig.1). This review surmises the superiority and the preparation method of AuNPs and concludes the general strategies of CL based on AuNPs for detection of biomolecules.

### The Outstanding Features of AuNPs

**Fig. 2:**
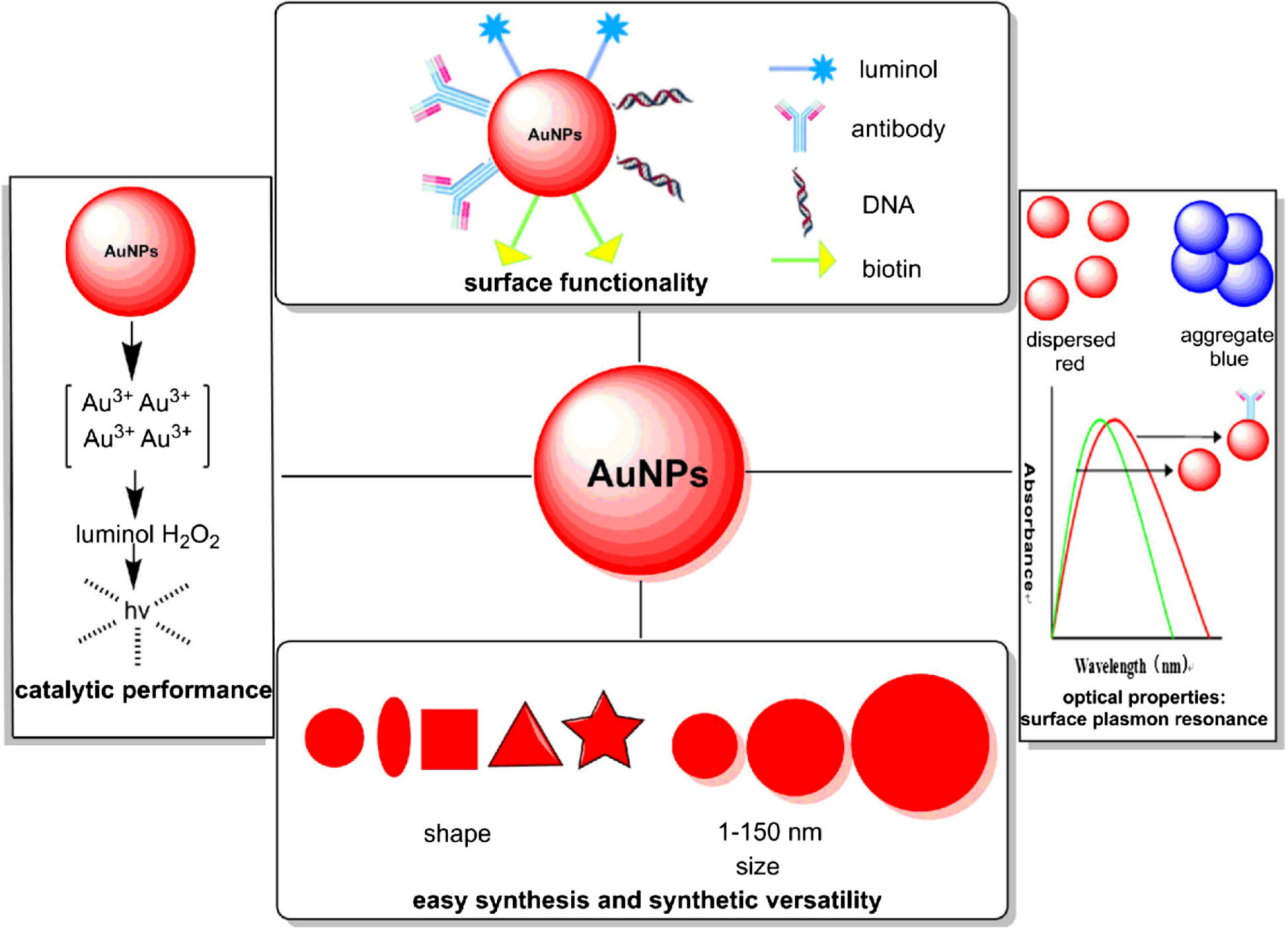
The unique attributes of AuNPs

AuNPs have some physical, chemical, optical, and electrical attributes [18] (Fig.2).

(i) Easy synthesis and synthetic versatility: AuNPs could be made in precise control over shape (sphere, rod, nanoshell, nanostar, triangular, nanocage) and size (1–150 nm) [15, 19–21]. The properties of AuNPs can be tuned by regulating the diameter and the shape because different shape and size has its distinct features [22, 23]. Therefore, we can make the right AuNPs to satisfy the applications at hand.
(ii) Surface functionality: the biocompatibility of AuNPs is excellent. Thiols and amines could bind to the surface of AuNPs so as to provide a facile way for some active group such as protein, biotin, peptide, and nucleic acids to conjugate with AuNPs by the chemistry of thiol-gold, gold-amine, or electrostatic interactions [24–26].
(iii) Chemical inert: AuNPs are essentially inert and do not cause acute cytotoxicity [27].
(iv) Catalytic performance: AuNPs, the diameter vary from 6 to 99 nm especially for 38 nm, can catalyze the system of luminol-H_2_O_2_ [28]. In addition, it also can directly catalyze the system of AgNO_3_-luminol.
(v) Optical properties: surface plasmon resonance (SPR) could detect the changes in nanoparticle aggregation states [29]. The color of dispersed AuNP solution appears red, while the aggregate solution appears blue for the reason that SPR shows the absorption maximum at longer wavelength, which is known as red-shift. If the size and the shape of AuNPs have changed, the peak of absorbance would be correspondingly changed.
(vi) Small size and high surface area to volume ratios: AuNPs could be loaded with plenty of biological reagents, and it can be made rather small so as to provide a high surface area to volume ratio, which maximize the payload/carrier ratio [29, 30].

### The Synthesis Method of AuNPs

**Fig. 3:**
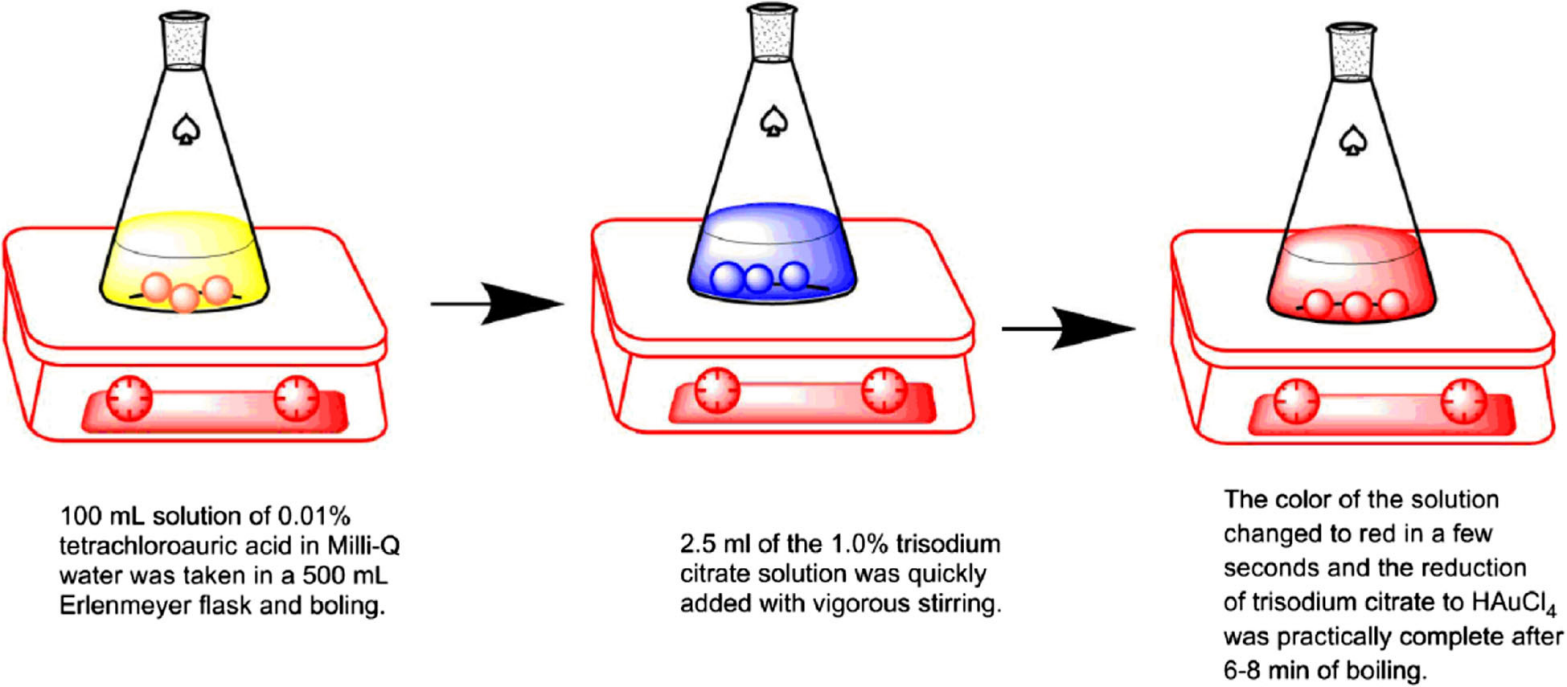
Reduction of HAuCl_4_ with sodium citrate in water: the sizes of AuNPs are depended on the concentration of sodium citrate

AuNPs can be synthesized by reduction of HAuCl_4._ The methods include chemical reduction, irradiation reduction [31, 32], sonochemical reduction [33], and biochemistry reduction [34, 17]. The most common way is the chemical reduction. In this reduction system, there must be reducing agent to reduction HAu^III^Cl_4_ to Au^0^ (10–150 nm) [35] and capping agent to bind to the surface of AuNPs with the purpose of controlling the size and inhabiting particle aggregation. The reducing agents include sodium citrate, sodium borohydride (NaBH_4_), and diborane. The corresponding capping agents are citrate, alkanethiol, and phosphine [36]. Among various synthesis methods for AuNPs, the method of citrate reduction is most widely used and the procedure is as followed (Fig. 3).

### The Strategies of CL Based on Labeling AuNPs

**Fig. 4:**
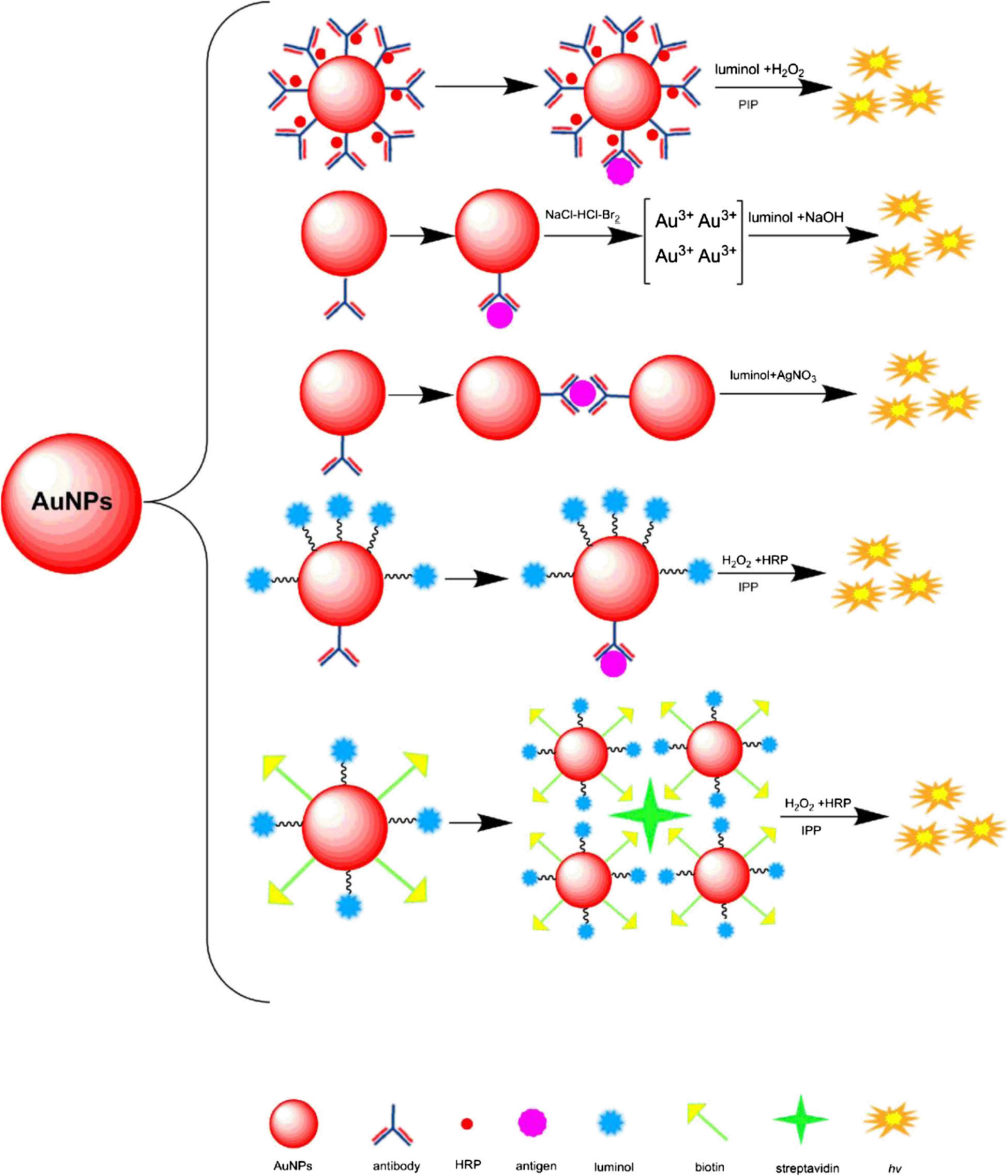
The strategies of CL based on labeling AuNPs

The CL detection methods based on AuNPs have two approaches: (i) AuNPs are directly used as catalyst to amplify CL signal. (ii) The detection signal is amplified by the biomolecules loading on the surface of AuNPs such as antibody, horseradish peroxidase (HRP), luminol, and biotin. The strategies are depicted as followed (Fig. 4).

In conventional CL detection, the AuNPs were dissolved with the solution of NaCl-HCl-Br_2_ to form AuCl_4_^−^, which could catalyze luminol to initial CL reaction. However, each AuNP contains thousands of gold atoms, and the NH_2_OH can catalyze AuNPs to form gold atoms. In such a condition, more AuCl_4_^−^ can be formed in NaCl-HCl-Br_2_ solution, leading to a high catalyze ability. Zhang et al. [37] developed a gold(III)-enhanced CLIA for detection of porcine circovirus (PCV). It was demonstrated that the detection signal could be further enhanced by NH_2_OH amplification assay so that the detection limit was lowered to 2.67 × 10^2^ copies mL^−1^.

The dissolution of AuNPs must be conducted under extremely severe conditions, which need a long time and complex operations, leading to a high CL background signal. Li et al. [38] used silver deposition on gold labels for detection of human immunoglobulin G (IgG). Although the sensitivity was further elevated, time-consuming was also a big drawback. Afterwards, non-stripping CL was developed, and it can shorten detection time greatly, which attracts researcher’s interests. Wang et al. [39] found that irregular AuNPs have a stronger catalytic efficiency than spherical AuNPs, but the complicated synthesis processes restrict its clinical application. Subsequently, luminol-AgNO_3_-AuNPs CL system was developed [40]. The mechanism of this system is that luminol is a reducing agent for reduction of AgNO_3_ to Ag atom; meanwhile, luminol was oxidized to luminol radicals under the catalysis of AuNPs. So, the normal spherical AuNPs could catalyze luminol to produce CL in the presence of AgNO_3_. According to this condition, Luo et al. [41] found an interesting phenomenon that the dispersed AuNPs can catalyze luminol-AgNO_3_ to produce a weak CL, while the aggregated AuNPs can produce a strong CL. Based on this finding, they developed a homogenous CLIA for determination of human IgG. The antibody-functionalized AuNPs could aggregate along with the immunoreaction of antigen and antibody, and the CL signal is enhanced obviously owing to the high catalytic activity of aggregated AuNPs. This method omitted washing and separation steps; the detection limit of IgG is as low as 3 pg mL^−1^.

Zhou et al. [42] developed a CL method using HRP and detection antibody-coated AuNPs as nanoprobes for detection of antibody against porcine parvovirus (PPV). After the nanoprobes captured the primary antibody, the solution of luminol and H_2_O_2_ were added immediately. The CL signal is amplified extremely because there are lots of HRP on the surface of AuNPs. In this assay, standard positive serum at the 1:1024 dilutions was still detected. Yang et al. [43] developed a CLIA detection method for α-fetoprotein (AFP) utilizing double-codified gold nanoparticles (DC-AuNPs) as probe and 4-(4-iodo)phenylphenol (IPP) as enhancer. The primary anti-AFP antibody was immobilized on the surface of magnetic beads (MB), and the AuNP was conjugated with both anti-AFP antibody and HRP. First, the antigen was captured with functional magnetic beads, and then, the immunocomplex were recognized by the DC-AuNPs. The detection limit is as low as 5 pg mL^−1^.

Yang et al. [44] developed a CLIA for detection of carcinoembryonic antigen based on AuNPs. The assay coimmobilizes not only secondary antibody but also luminol onto the AuNPs. 3-MPA was used as a linker to connect with AuNPs via sulfur-gold bond firstly, and then, the secondary antibody and luminol was conjugated with 3-MPA. Magnetic beads were used as carriers to loading primary antibody. HRP and Co^3+^ were used as catalysts. The detection limit for CEA is as low as 5.0 × 10^−11^ and 1.0 × 10^−10^ gmL^−1^ correspondingly.

The coimmobilization antibody and luminol onto the surface of AuNPs result in a lower CL signal than luminol free in the solution, which attributes to the change in charge, steric hindrance accessibility, and the structure of luminol. In order to overcome this drawback, Shourian et al. [45] replaced antibodies with biotin to immobilization on the surface of AuNPs. The AuNP-biotin-luminol and AuNP-antibody-luminol system were compared, and the results showed that the former signal was 12 times higher than the latter, because the antibody occupied most of the surface and restricted the connection of luminol. On the contrary, the size of biotin is so small that luminol could attach to the AuNPs sufficiently. They applied biotin-streptavidin reaction in CLIA based on AuNPs for detection of hepatitis B surface antigen. The assay loading biotin and luminol molecule on the surface of AuNPs and the secondary antibody was modified with streptavidin. After secondary antibody recognized the primary antibody, the biotin-luminol-AuNPs would bond to the immunocomplex by the reaction of biotin-streptavidin. Streptavidin is a tetrameric protein so that more than one biotin molecule would bond to it. In addition, the binding constant of biotin-streptavidin is high and the complex has a high stability in a wide pH and temperature. All of these benefits make this system a very high-sensitivity assay, with a detection limit of 0.358 pg mL^−1^ for hepatitis B surface antigen.

### Label-Free CL Detection Method by the Catalytic of AuNPs

**Fig. 5:**
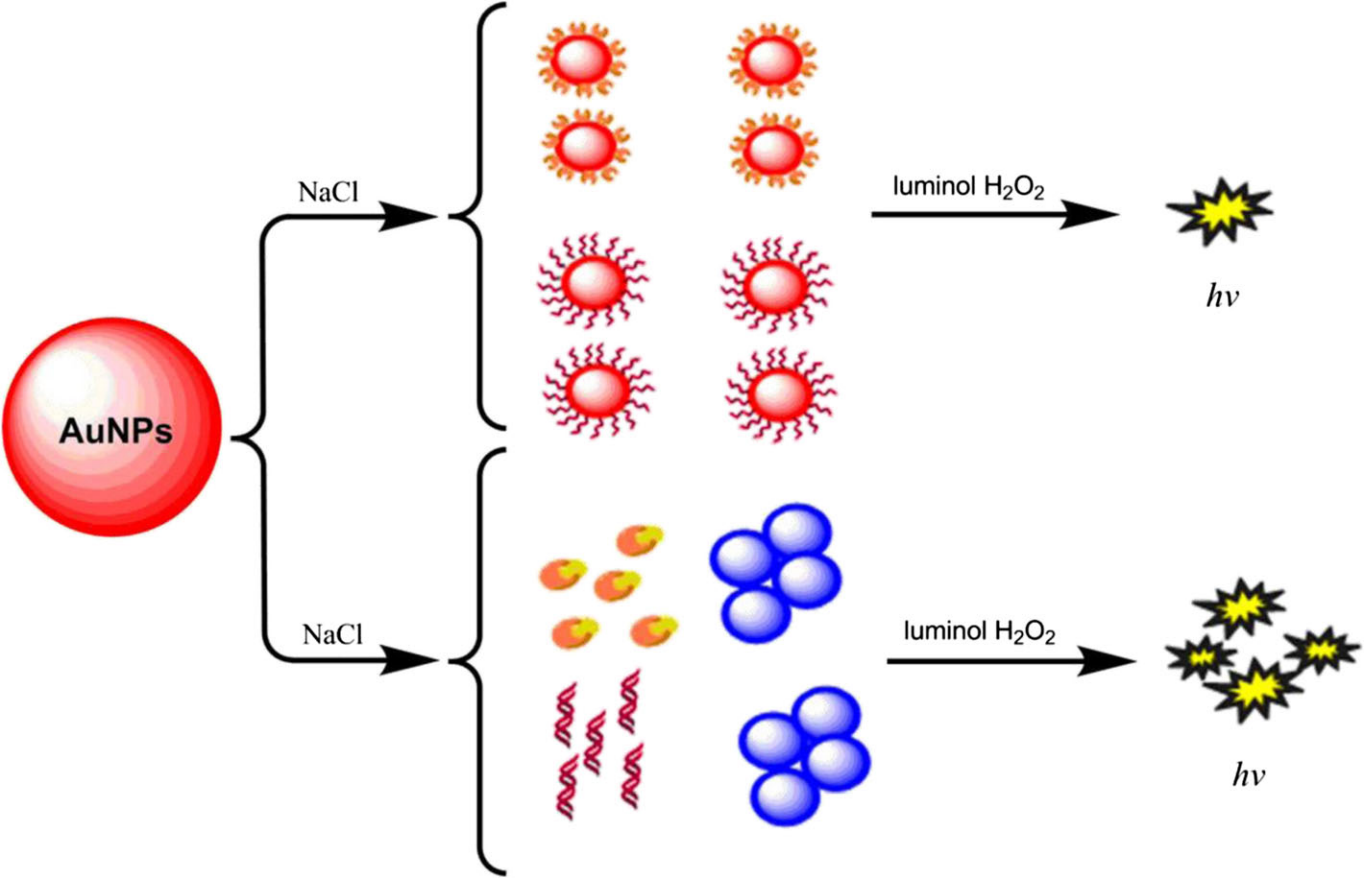
The strategies of label-free CL based on AuNPs

Labeling biomolecules such as antibody, HRP, and luminol are very troublesome. Therefore, label-free AuNP-initialed CL detection method was developed recently (Fig. 5).

Regularly, in the aqueous solution of AuNPs, there must be capping agents to inhibit the aggregation of nanoparticles. But in 2004, an interesting phenomenon that a certain concentration salt can screen the repulsion between AuNPs and make aggregation of them was reported [46]. The aggregated AuNPs could initial a stronger CL than dispersed in the system of luminol-H_2_O_2_. Based on these findings, Islam and Kang [47] developed a label-free CL method for detection of C-reactive protein (CRP). The ligand of CRP is O-phosphorylethanolamine (PEA), which has a distinct property that can stick to the surface of gold so that it can inhibit the aggregation of AuNPs in the presence of salt and initial a weak CL. Once the reaction of CRP-PEA happened, the AuNPs would aggregate and show a strong CL signal. This CL method does not require labeling and strict stripping procedures; the detect limit is as low as 1.88 fM.

Qi et al. [48] developed a label-free DNA hybridization method based on the AuNP-initialed CL. The principle is that AuNPs could not aggregate in the presence of single-strand DNA (ss-DNA) probe and 0.5 M NaCl for the reason that ss-DNA absorb onto the surface of AuNPs and inhibit the aggregation of them. After the target DNA was added, the ss-DNA probe would be removed by the reaction of hybridization, leading to the aggregation of AuNPs and making a stronger CL. The detection limit is as low as 1.1 fM.

In a similar vein, Zhang et al. [49] developed a label-free CL method for detection of fibrillar fibrin. The fibrinogen could absorb on the surface of AuNPs and inhibit the aggregation of nanoparticles in the presence of 1 M NaCl. After thrombins catalyze fibrinogen to fibrillar fibrin, the AuNPs would aggregate and initial a stronger CL. Based on this principle, the limit of detection for fibrin was as low as 1 fM.

In the system of luminol-H_2_O_2_, some active groups such as −NH_2_, −SH could struggle for active oxygen intermediates with luminol, making the decrease of CL signal [28]. Liu et al. [50] found that l-cys could also inhibit the CL of luminol-H_2_O_2_-AuNP system. Therefore, they integrate laminated paper-based analytica device (LPAD) with AuNP-catalyzed CL method for determination of l-cys. This detection system not only has the superiority of LAPD such as low cost and suitable for point of care (POC) but also has the high sensitivity of CL. The detection limit is as low as 8.2 × 10^−10^ M.

### CLIA Based on AuNPs Couple with Separation Technique

It seems that the usual CLIA has multifarious washing and separating steps, which is time-consuming and sophisticated. Recently, capillary electrophoresis (CE), high-performance liquid chromatography (HPLC), and flow injection (FI) coupled with CLIA have attracted great interests for the merits of high separation efficiency, ease of automation, and time-saving. Jiang et al. [51] combined CE with AuNP-amplified CL to detection of carcinoembryonic antigen (CEA). The CEA antibody and HRP were also conjugated with AuNPs. After the nanoprobes recognized the antigen, the excess AuNP conjugates were separated by the CE, and the CL was activated by the p-iodophenol (PIP)-enhanced luminol-H_2_O_2_-HRP system. The detection process could be finished within 5 min, and the detection limit is as low as 0.034 ng mL^−1^. Liu et al. [52] proposed a novel protocol for detection of protein by means of aptamer-functionalized AuNPs and CE-CL. Taking thrombin as a model, they linked thrombin-binding aptamer to the AuNPs by the SH–Au covalent bond and blocked the spare binding side with blocker DNA. After CE separated, luminol and H_2_O_2_ were added. The detection limit is down to 13.5 fmol L^−1^.

Li et al. [53] found that some reductive compounds such as monoamine neurotransmitters and their metabolites have an inhibitory effect on the luminol-AgNO_3_-AuNPs. These reductive compounds have a stronger reductive ability than luminol, and competed with luminol for AgNO_3_, making that the generation of luminol radicals is decreased and leading to a weak CL signal. By taking advantage of this phenomenon, they developed a CL method for simultaneous determination of monoamine neurotransmitters and their metabolites in a mouse brain microdialysate. HPLC was applied in this system, and the method was simple, sensitive, and fast.

Hao and Ma [54] combined FI system with AuNP-enhanced CL to detection of carcinoembryonic antigen (CEA). They loaded the secondary antibody onto the surface of AuNPs and utilized the method of dissolving AuNPs into HNO_3_-HCl solution. The detection limit is as low as 20 pg mL^−1^.

## Conclusions

CL as the newest labeling technique has been widely applied in biomedical diagnosis during the two decade. Nanodiagnostic techniques especially for AuNPs have gained much attention by the researchers. Coupling both techniques to detection of biological agents is a trend in the last several years. Continuous efforts for labeling biomarkers (e.g. proteins, genes, and chemiluminescent agent) onto the surface of AuNPs are made so as to improve the sensitivity of CL. The labeling strategies are improving constantly, and the detection limit is becoming lower. In addition, the separation technique and the label-free strategies are also developed quickly because it can simplify the operation and save more time. All of these efforts are to develop rapid, sensitive, automatic, and point of care detection methods. Currently, some new and readily contagious viruses such as Zika virus, Ebola virus, and avian influenza virus, which could pose a great disaster for human, have not been detected in a quick and sensitive way. If the CL based on AuNP detection method could be developed for those virus detection, the significance for public health would be important. In the next 20 years, the CL based on AuNP detection method would become the most widely used detection method in clinical medicine and clinical veterinary science.
